# Sleep duration and the risk of breast cancer: the Ohsaki Cohort Study

**DOI:** 10.1038/sj.bjc.6604684

**Published:** 2008-09-23

**Authors:** M Kakizaki, S Kuriyama, T Sone, K Ohmori-Matsuda, A Hozawa, N Nakaya, S Fukudo, I Tsuji

**Affiliations:** 1 Division of Epidemiology, Department of Public Health and Forensic Medicine, Tohoku University Graduate School of Medicine, Sendai, Japan; 2 Division of Behavioral Medicine, Department of Functional Medical Science, Tohoku University Graduate School of Medicine, Sendai, Japan

**Keywords:** sleep duration, breast cancer, incidence, Japanese, prospective cohort study

## Abstract

In a prospective study of 23 995 Japanese women, short sleep duration was associated with higher risk of breast cancer (143 cases), compared with women who slept 7 h per day, the multivariate hazard ratio of those who slept ⩽6 h per day was 1.62 (95% confidence interval: 1.05–2.50; *P* for trend=0.03).

Breast cancer is the commonest cancer in women, worldwide (Parkin *et al*, 2005). In Japan, the incidence rate age-standardised to the world population was 28.3 per 100 000 in 1991 and 39.5 in 2001 (The Research Group for Population-based Cancer Registration in Japan, 1998; Marugame *et al*, 2007).

Melatonin, which is secreted mainly from the pineal gland and plays a role in sleep duration, is suggested as an agent in the association between sleep duration and breast cancer (Brzezinski, 1997; Schernhammer and Schulmeister, 2004). This is because melatonin suppresses the synthesis and secretion of sex hormones by promoting the release of gonadotropin-releasing hormone (Martin and Klein, 1976; Aleandri *et al*, 1996). In relation to melatonin secretion, there have been several observational studies on night work or visual impairment and breast cancer (Feychting *et al*, 1998; Verkasalo *et al*, 1999; Kliukiene *et al*, 2001; Schernhammer *et al*, 2001; Megdal *et al*, 2005; Pukkala *et al*, 2006; Schwartzbaum *et al*, 2007). In addition, there have been three prospective cohort studies of sleep duration and the risk of breast cancer, although with inconsistent findings (Verkasalo *et al*, 2005; Pinheiro *et al*, 2006; Wu *et al*, 2008).

We therefore examined the association between sleep duration and the risk of breast cancer in a population of Japanese women.

## Materials and methods

Details of the Ohsaki National Health Insurance (NHI) Cohort Study have been described previously (Tsuji *et al*, 1998; Kuriyama *et al*, 2006). Briefly, this prospective cohort study, started in 1994, included 28 515 women aged 40–79 years living in the 14 municipalities of Miyagi Prefecture, northeastern Japan. The response rate was 95.0% (*N*=27 134) for the questionnaire, including items on sleep duration and other health-related lifestyle factors. The study protocol was reviewed and approved by the ethics committee of Tohoku University School of Medicine.

After exclusion of participants who had withdrawn from the NHI before follow-up, those who had history of cancer, those who had omitted responses for sleep duration, and those who had reported sleep duration of less than 4 h or more than 12 h, 23 995 participants remained. To follow up participants for mortality and migration, we reviewed the NHI withdrawal history files for 1995–2003. Through the Miyagi Prefectural Cancer Registry, we identified 143 incident cases of breast cancer.

With regard to the sleep duration, participants answered the mean integer number of hours of sleep per day during the last year. Because of the small number who slept for less than 7 h and less than 8 h, we categorised sleep duration into four groups: ⩽6, 7, 8, and ⩾9 h per day. We estimated hazard ratios (HRs) and 95% confidence intervals (CIs) of breast cancer incidence according to sleep duration, using the Cox proportional hazards model, with adjustment for age and potential confounders. The continuous *P* for trend was calculated by treating sleep duration as a continuous variable, and the categorical *P* for trend by treating each category as a continuous variable. Interactions between the risk and all confounders were tested through the addition of cross-product terms to multivariate model.

All statistical analyses were performed using SAS statistical software, version 9.1 (SAS Institute Inc, Cary, NC, USA), and all those reported were two-sided; differences at *P*-values of <0.05 were accepted as significant.

## Results

Table 1 shows the baseline characteristics of participants according to sleep duration. Participants who slept 6 h or less were more likely to have a family history of cancer, to have used oral contraceptive drugs, and to be premenopausal. Participants who slept 9 h or more were older, had a smaller total caloric intake, lower educational level, were more likely to have a history of diseases, and were less likely to be employed, married, and premenopausal.

Using women who slept 7 h as the reference group, we found an inverse association between sleep duration and breast cancer risk. The HR of women who slept 6 h or less was 1.62 (95% CI: 1.05–2.50), of those who slept 8 h was 1.14 (95% CI: 0.75–1.73), and of those who slept 9 h or more was 0.72 (95% CI: 0.36–1.43) (*P* for trend=0.03). This result did not change substantially when participants whose event occurred within 3 years of baseline (*N*=49) were excluded and stratified analysis by age and menopausal status (Table 2). In addition, we examined in detail confounding and effect modification by other covariates on the associations between sleep duration and the risk of breast cancer. No statistically significant interaction was observed between sleep duration and other confounding factors for the risk of breast cancer on a multiplicative scale (data not shown).

## Discussion

This study revealed an inverse association between sleep duration and the risk of breast cancer in Japanese women, participants who slept 6 h or daily having a significantly increased risk of breast cancer.

There have been three prospective cohort studies of breast cancer in relation to sleep duration (Verkasalo *et al*, 2005; Pinheiro *et al*, 2006; Wu *et al*, 2008), of which the last two reported a significantly decreased risk in long sleepers and our results are consistent with these. By contrast, another study reported no such association (Pinheiro *et al*, 2006), possibly studied because residential nurses were studied with rotating-shift work and varying timing of sleep, so that generalising from their results may be inappropriate.

Melatonin is suggested to be involved in this relationship with sleep duration, a decrease that results in a shorter duration of nocturnal melatonin secretion (Wehr, 1991). A lower melatonin level was associated with an increased risk of breast cancer (Schernhammer and Hankinson, 2005; Schernhammer *et al*, 2008). Melatonin may have an inhibitory effect on gonadal function, including the synthesis and secretion of sex hormones, by promoting the release of gonadaotropin-releasing hormone (Martin and Klein, 1976; Aleandri *et al*, 1996); it also exerts an antiproliferative effect on breast cancer cell lines (Blask *et al*, 1997).

Our study had several strengths. First, we recruited participants from the general population, allowing possible generalisation of our results. Second, the Miyagi Prefectural Cancer Registry is one of the earliest and most accurate population-based cancer registries in Japan (Takano and Okuno, 1997), with only 2.7% of breast cancer cases ascertained by death certificate only (DCO) in 1998–2002 (Curado *et al*, 2007).

Our study also had several methodological limitations. First, we used self-reported sleep duration, and the assessment was done only once. Second, we had no information on such factors as sleep quality, the timing of sleep, the use of sleep medication, or the presence of sleeping disorders that can influence sleep duration and thereby might affect breast cancer risk. Finally, we had no information about rotating-shift work or night work, but since 23% of our participants were housewives, 19.0% farmers, and 15.7% retired, such details would have been unlikely to have changed the result substantially.

In conclusion, we have found a significant inverse association between sleep duration and breast cancer risk in Japanese women, those who slept 6 h or less having a significantly increased risk.

## Figures and Tables

**Table 1:** Baseline characteristics of the participants according to sleep duration

	**Sleep duration (hours per day)**			
	**⩽6**	**7**	**8**	**⩾9**
Number of participants	4549	7087	8667	3692
Mean age (years), s.d.^a^	58.8 (10.5)	58.3 (10.0)	61.4 (9.3)	66.4 (8.7)
Mean body mass index (kg m^−2^), s.d.^a^	23.6 (3.3)	23.7 (3.3)	23.8 (3.4)	24.0 (3.9)
Mean total caloric intake (kcal day^−1^), s.d.^a^	1220.2 (378.8)	1263.0 (367.7)	1242.1 (380.0)	1171.8 (410.6)
				
*History of diseases (%)* b				
Presence	29.9	28.7	34.2	44.2
Absence	70.1	71.3	65.8	55.8
				
*Family history of cancer in first-degree relatives (%)*				
Presence	35.0	33.1	31.1	29.7
Absence	65.0	66.9	68.9	70.3
				
*Job (%)*				
Employed	35.2	38.8	32.6	23.5
Unemployed	38.0	36.1	39.5	43.6
				
*Marital status (%)*				
Married	66.0	70.5	66.8	54.8
Unmarried	23.8	18.9	20.0	27.3
				
*Education (%)*				
Junior high school or less	46.9	46.8	57.6	66.3
High school	36.8	39.0	29.6	18.2
College/university or higher	10.4	9.0	6.2	3.4
				
*Cigarette smoking (%)*				
Never smoker	69.1	72.2	68.9	65.3
Ex-smoker	2.7	1.8	1.8	2.2
Current smoker (<20 cigarettes per day)	5.7	4.2	3.3	2.8
Current smoker (⩾20 cigarettes per day)	3.2	2.1	1.4	1.1
				
*Alcohol consumption (%)*				
Never drinker	54.7	60.0	60.2	60.3
Ex-drinker	4.7	3.3	3.1	4.1
Current drinker	25.3	21.0	16.2	12.1
				
*Walking status (%)*				
Longer than 1 h per day	38.5	39.7	38.6	36.0
Less than 1 h per day	52.3	51.5	51.2	51.3
				
*Menopausal status (%)*				
Premenopausal	23.7	23.5	13.9	5.7
Postmenopausal	62.5	62.5	68.2	68.1
				
*Age at menarche (%)*				
⩽13 years	7.0	7.4	6.3	5.4
14–5 years	19.5	20.5	20.7	17.0
⩾16 years	18.1	16.7	19.6	22.2
				
*Age at first delivery (%)*				
⩽21 years	16.3	16.0	18.3	20.6
22–5 years	48.4	52.1	50.6	45.2
⩾26 years	20.6	18.8	16.1	13.1
				
*Number of deliveries (%)*				
0 births	3.1	2.7	2.8	3.0
1– births	39.3	40.0	35.3	22.4
⩾3 births	43.6	45.4	47.2	52.7
				
*Using of oral contraceptive drugs (%)*				
Yes	5.1	4.4	3.1	2.5
No	81.0	82.5	79.2	72.9
				
*Using of hormone drugs except for oral contraceptive drugs (%)*				
Yes	8.1	6.8	6.3	5.7
No	77.9	79.7	76.4	69.3

a standard deviation.

b History of stroke, hypertension, myocardial infarction, or diabetes mellitus.

**Table 2:** Cox proportional hazard ratios (HRs) and 95% confidence intervals (CIs) for breast cancer incidence according to sleep duration in Japanese women

	**Sleep duration (hours per day)**					
	**⩽6**	**7**	**8**	**⩾9**	*P* for trend*	***P* for trend^†^**
*All-cases*						
Person-Year	35 208	55 574	67 368	27 715		
Number of event	42	40	50	11		
Crude HR (95% CI)	1.66 (1.08–2.56)	1.00 (reference)	1.03 (0.68–1.56)	0.55 (0.28–1.07)	0.002	0.001
Age-adjusted HR (95% CI)	1.67 (1.08–2.58)	1.00 (reference)	1.09 (0.71–1.65)	0.63 (0.32–1.24)	0.006	0.006
Multivariable HR (95% CI)^a^	1.63 (1.06–2.52)	1.00 (reference)	1.14 (0.75–1.73)	0.71 (0.36–1.41)	0.03	0.02
Multivariable HR2 (95% CI)^b^	1.62 (1.05–2.50)	1.00 (reference)	1.14 (0.75–1.73)	0.72 (0.36–1.43)	0.03	0.03
Multivariable HR3 (95% CI)^c^	1.67 (1.002–2.78)	1.00 (reference)	0.99 (0.59–1.65)	0.29 (0.09–0.98)	0.002	0.002
						
*Stratified analysis*						
Age (*P* for interaction=0.48)						
<60 years						
Person-Years	17 048	27 696	25 216	5666		
No. of cases	26	21	22	2		
Multivariable HR (95% CI)^b^	2.01 (1.12–3.59)	1.00 (reference)	1.16 (0.63–2.13)	0.43 (0.10–1.87)	0.02	0.02
⩾60 years						
Person-Years	18 160	27 878	42 152	22 049		
No. of cases	16	19	28	9		
Multivariable HR (95% CI)^b^	1.20 (0.61–2.35)	1.00 (reference)	1.09 (0.61–1.97)	0.81 (0.36–1.82)	0.46	0.48
Menopausal status (*P* for interaction=0.70)						
Premenopausal						
Person-Years	8154	12 907	9286	1617		
No. of cases	11	8	9	0		
Multivariable HR (95% CI)^b^	2.06 (0.81–5.23)	1.00 (reference)	1.48 (0.56–3.93)	—	0.10	0.27
Postmenopausal						
Person-Years	22 331	35 004	46 183	18 933		
No. of cases	28	29	35	9		
Multivariable HR (95% CI)^b^	1.46 (0.86–2.46)	1.00 (reference)	1.04 (0.63–1.70)	0.74 (0.35–1.59)	0.11	0.09

^*^P for trend values were calculated by treating sleep duration as a continuous variable.

^†^*P* for trend values were calculated by treating each categories of sleep duration as a continuous variable.

a Multivariable HR was adjusted for age (continuous variable); body mass index (<18.5, 18.5–25.0, ⩾25.0 kg m^−2^); history of diseases (having history of stroke, hypertension, myocardial infarction, or diabetes mellitus); family history of cancer (presence or absence in first-degree relatives); job (employed or unemployed); marital status (married or unmarried); education (junior high school or less, high school, or college/university or higher); cigarette smoking (never smoker, ex-smoker, current smoker 1–19 cigarettes per day, or current smoker ⩾20 cigarettes per day); alcohol consumption (never drinker, ex-drinker, or current drinker); time spent walking (less than 1 h per day, or longer than 1 h per day).

b Multivariable HR2 was adjusted for above plus total caloric intake (continuous variable, kcal day^−1^); menopausal status (premenopausal or postmenopausal); age at menarche (⩽13 years, 14–15 years, or ⩾16 years); age at first delivery (⩽21 years, 22–25 years, or ⩾26 years); number of deliveries (0 births, 1–2 births, or ⩾3 births); using of oral contraceptive drugs (yes or no); using of hormone drugs except for oral contraceptive drugs (yes or no).

c Multivariable HR3 denotes the HR2 with cases diagnosed in the first 3 years of follow-up excluded from the analysis.
